# The genus *Paleosepharia* Laboissière, 1936 in Taiwan: review and nomenclatural changes (Coleoptera, Chrysomelidae, Galerucinae)

**DOI:** 10.3897/zookeys.744.22970

**Published:** 2018-03-19

**Authors:** Chi-Feng Lee

**Affiliations:** 1 Applied Zoology Division, Taiwan Agricultural Research Institute, 189 Chung-Cheng Road, Wufeng, Taichung 413, Taiwan

**Keywords:** Food plants, leaf beetles, new combination, taxonomic revision

## Abstract

Taxonomic study of species of the genus *Monolepta* Chevrolat, 1836 with subscutellar incised ridges in males and occurring in Taiwan resulted in the transfer of all species to *Paleosepharia* Laboissière, 1936: *P. amiana* (Chûjô, 1962), **comb. n.**, *P. formosana* (Chûjô, 1935), **comb. n.**, *P. nantouensis* (Kimoto, 1996) **comb. n.**, and *P. yasumatsui* (Kimoto, 1969), **comb. n.** The position of *M. excavata* Chûjô, 1938 in *Paleosepharia* is confirmed. Lectotypes are designated for *M. excavata* Chûjô, 1938 and *M. formosana* Chûjô, 1935. Generic characters of *Paleosepharia* are re-evaluated in the context of these nomenclatural changes.

## Introduction

Members of the genus *Paleosepharia* Laboissière resemble those of *Monolepta* Chevrolat, but both genera can be separated externally by specific structures of the elytral epipleuron and the sexually dimorphic elytra. The epipleuron is abbreviated or suddenly narrowed before the middle, and subscutellar bulges or incised ridges are absent from the elytra in both sexes of *Monolepta*. In contrast the epipleuron continues to the apex, and subscutellar bulges or incised ridges are present on the elytra in males of *Paleosepharia* (Mohamedsaid 1996; Wagner and Bieneck 2012; Medvedev 2014; Rizki et al. 2014, 2016). In addition, some additional genitalic characters were proposed for *Paleosepharia* by Rizki et al. (2016). Taxonomic status of some species of *Monolepta* should be re-evaluated in cases where they were described based only on female specimens because important sexually dimorphic diagnostic characters may have been excluded. *Paleosepharia* comprises 76 species and one subspecies in the Oriental and Palaearctic regions (Nie et al. 2017)

Among the 30 species of *Monolepta* described from Taiwan so far (Kimoto and Takizawa 1997), *M. excavata* Chûjô, 1938 was transferred *Paleosepharia* by Gressitt and Kimoto (1963), but that transfer was not followed by subsequent taxonomists except Beenen (2010). Some species of *Monolepta* in Taiwan could be members of *Paleosepharia* but their diagnostic characters are unclear due to insufficient material. Extensive collections have been conducted by the Taiwan Chrysomelid Research Team (TCRT) since 2007, resulting in the publication of several books and many taxonomic and nomenclatural changes, including the study reported here (Lee and Cheng 2007, 2010; Lee et al. 2016). Five Taiwanese species of *Monolepta* possess subscutellar incised ridges in males, including *M. amiana* Chûjô, 1962, *M. excavata* Chûjô, 1938, *M. formosana* Chûjô, 1935, *M. nantouensis* Kimoto, 1996, and *M. yasumatsui* Kimoto, 1969. In the study reported here, the status of these species are revaluated and genitalic characters proposed by Rizki et al. (2016) are compared with other members of the genus and those of *Paleosepharia*.

## Materials and methods

The abdomens of adults were separated from the forebody and boiled in 10 % KOH solution, followed by washing in distilled water to prepare genitalia for illustrations. The genitalia were then dissected from the abdomen, mounted on slides in glycerin, and studied and drawn using a Leica M165 stereomicroscope. For detailed examinations a Nikon ECLIPSE 50i microscope was used.

At least three pairs from each species were examined to delimit variability of diagnostic characters. For species collected from more than one locality, at least one pair from each locality was examined. Length was measured from the anterior margin of the eye to the elytral apex, and width at the greatest width of the elytra.

Specimens studied herein are deposited at the following institutes and collections:

**KMNH** Kitakyushu Museum of Natural History and Human History, Kitakyushu, Japan [Yûsuke Minoshima];

**KUEC** Faculty of Agriculture, Kyushu University, Fukuoka, Japan [Osamu Tadauchi];

**NMNS** National Museum of Natural Science, Taichung, Taiwan [Ming-Luen Jeng];

**SDEI** Senckenberg Deutsches Entomologisches Institut, Müncheberg, Germany [Kostantin S. Nadein];

**TARI** Taiwan Agricultural Research Institute, Taichung, Taiwan

Exact label data are cited for all type specimens of described species; a double slash (//) divides the data on different labels and a single slash (/) divides the data in different rows. Other comments and remarks are in square brackets: [p] – preceding data are printed, [h] – preceding data are handwritten, [w] – white label, [y] – yellow label, [b] – blue label, and [r] – red label.

## Systematics

### Paleosepharia amiana

Taxon classificationAnimaliaColeopteraChrysomelidae

(Chûjô) comb. n.

Figs 1A–1C , 2

Monolepta amiana Chûjô, 1962: 136 (Taitung); Kimoto 1969: 47 (Nantou); Kimoto 1991: 13 (Kaohsiung); Kimoto and Chu 1996: 78 (catalogue); Kimoto and Takizawa 1997: 385 (catalogue); Beenen 2010: 482 (catalogue); Yang et al. 2015: 266 (catalogue); Lee et al. 2016: 108 (food plants).

#### Type material.

Depository of the single female holotype is unknown. Chûjô (1962) indicated that most specimens were deposited at the TARI except for some in his collection. Although some specimens were found at the KUEC, the type of this species was not among them.

**Other material examined (n = 82). Pingtung**: 1♀ (TARI), Jinshuiying [浸水營], 12.VIII.2010, leg. J.-C. Chen; 1♀, Tahanshan [大漢山], 4.X.2010, leg. J.-C. Chen; 6♀♀ (TARI), same locality, 22.IX.2011, leg. J.-C. Chen; 3♂♂, 11♀♀ (TARI), same locality, 3.IX.2012, leg. Y.-T. Chung; 1♂ (TARI), same locality, 13.IX.2012, leg. J.-C. Chen; 1♂, 1♀ (TARI), same locality, 14.IX.2012, leg. Y.-T. Chung; 1♂ (TARI), same locality, 30.VII.2013, leg. B.-X. Guo; 1♀ (TARI), same locality, 17.VIII.2013, leg. J.-C. Chen; 1♂, 2♀♀ (TARI), same locality, 3.IX.2013, leg. Y.-T. Chung; 7♀♀ (TARI), same locality, 11.IX.2013, leg. Y.-T. Chung; 2♂♂, 8♀♀ (TARI), same locality, 24.IX.2013, leg. Y.-T. Chung; 2♂♂, 3♀♀ (TARI), same locality, 1.X.2013, leg. Y.-T. Chung; 1♀ (TARI), same locality, 8.X.2013, leg. Y-T. Chung; 4♀♀ (TARI), same locality, 25.X.2013, leg. Y.-T. Chung; 1♀ (TARI), same locality, 16.XII.2013, leg. Y.-T. Chung; 2♂♂ (TARI), same locality, 19.VII.2014, leg. W.-C. Liao; 2♀♀ (TARI), same locality, 17.VI.2016, leg. Y.-T. Chung; 1♂ (TARI), same locality, 6.VIII.2016, leg. Y.-T. Chung; **Taipei**: 2♀♀ (TARI), Manyuehyuan [滿月圓], 3.VII.2010, leg. H. Lee; 1♀ (TARI), same but with “8.VII.2010”; 1♀ (TARI), same but with “8.IV.2010”; 1♀ (TARI), same but with “7.VIII.2014”; **Taitung**: 2♀♀ (TARI), Lichia trail [利嘉林道], 15.VII.2014, leg. Y.-T. Chung; 1♂ (TARI), same locality, 17.VII.2014, leg. Y.-T. Wang; 1♀ (TARI), same locality, 25.VII.2015, leg. Y.-T. Chung, P.-H. Kuo & S.-P. Wu; 1♂ (TARI), same locality, 1.VII.2016, B.-X. Guo; **Taoyuan**: 2♀♀ (TARI), Hsiaowulai [小烏來], 29.IX.2009, leg. M.-H. Tsou; 4♀♀ (TARI), same locality, 10.X.2009, leg. M.-H. Tsou; 1♂, 1♀ (TARI), Sankuang [三光], 14.VII.2010, leg. H. Lee.

#### Diagnosis.

Members of *Paleosepharia amiana* are similar to those of *P. excavata*, *P. formosana*, and *P. yasumatsui* with black stripes along the outer margins of yellow elytra. However, this species is easily recognized by the presence of only one transverse black band on the elytra (two transverse bands in others). The aedeagus of male *P. amiana* is similar to that of *P. nantouensis* in possessing a relatively narrow penis (more than 6.5 times longer than wide; less than 6.0 times in other species), acute apex of tectum (bifurcate apex in other species), and one pair of elongate and apically curved spiculae (lacking such spiculae in other species). It differs by the broader tectum (broader than penis; narrower than penis in *P. nantouensis*) and different sizes of the two pairs of hooked spiculae (same sizes in *P. nantouensis*).

#### Description.

**Figures 1.**
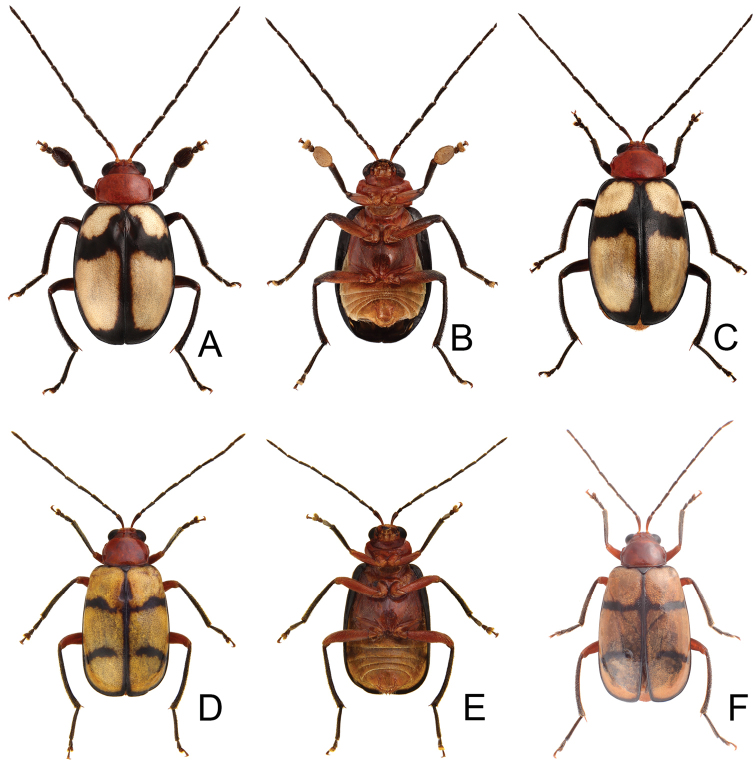
Habitus of *Paleosepharia* species. **A** *P. amiana* (Chûjô), male, dorsal view **B** Ditto, ventral view **C** *P. amiana* (Chûjô), female, color variation, dorsal view **D** *P. excavata* (Chûjô), male, dorsal view **E** Ditto, ventral view **F** *P. excavata* (Chûjô), female, dorsal view.

**Figures 2.**
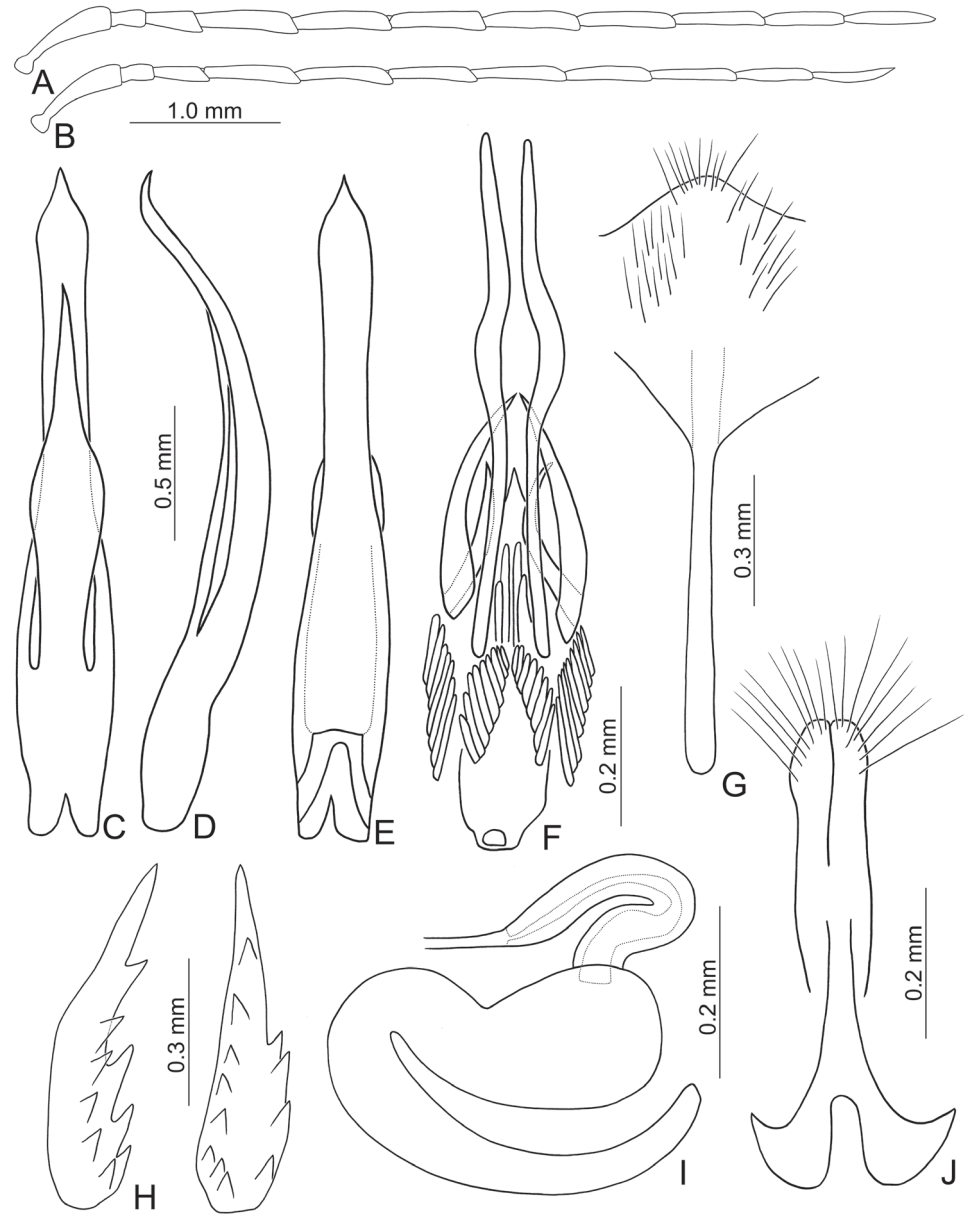
Diagnostic characters of *Paleosepharia amiana* (Chûjô). **A** Antenna, male **B** Antenna, female **C** Penis, dorsal view **D** Penis, lateral view **E** Penis, ventral view **F** Endophallic spiculae **G** Abdominal ventrite VIII **H** Bursa sclerites, left sclerite in lateral view, right sclerite in dorsal view **I** Spermatheca **J** Gonocoxae.

Males. Length 6.0–6.5 mm, width 3.3–3.4 mm. General color reddish brown (Fig. 1A–1B); antennae except two basal antennomeres, scutellum, tibiae, and tarsi black; elytra yellow, with wide black stripe along lateral margins and suture extending to apex, and one transverse, wide black band at basal 2/3. Antenna (Fig. 2A) filiform, ratio of length of antennomeres I to XI 1.0 : 0.3 : 0.5 : 0.9 : 0.9 : 0.9 : 0.9 : 0.9 : 0.8 : 0.7 : 0.8; ratio of length to width from antennomere I to XI 4.6 : 1.9 : 2.8 : 5.0 : 5.1 : 5.1 : 5.7 : 6.0 : 6.5 : 5.7 : 7.1. Pronotum 1.86–1.97 times wider than long; lateral margins slightly curved, basal margin slightly curved, apical margin slightly concave; disc with reticulate microsculpture and dense, minute punctures. Elytra 1.34–1.45 times longer than wide; lateral margins curved, widest behind middle; disc with one pair of oblique depressions starting from suture at scutellum, longitudinal ridge present inside each depression and along suture; disc with dense, minute punctures; apex truncate. First tarsomeres of front legs strongly swollen and dorso-ventrally flattened. Penis (Fig. 2C–E) extremely slender, 7.3 times longer than wide; parallel-sided, basally widened from middle, apically tapering; tectum elongate from basal 1/4 to apical 1/5, widest at midpoint and wider than penis, apically tapering, apex acute; moderately recurved at middle and near apex in lateral view; ventral surface with lateral areas membranous. Endophallic spiculae complex (Fig. 2F) with one pair of extremely elongate spiculae, apices curved at middle; two pairs of elongate hooked spiculae, one pair longer and curved inwards, the other pair shorter and curved outwards; one pair of longitudinal rows of elongate, apically curved setae near base; one row of elongate, apically tapering setae at sides behind middle.

Females. Length 5.6–6.5 mm, width 3.3–3.5 mm. Similar to male (Fig. 1C) but elytra lacking oblique depressions; first tarsomeres of front legs normal; ratio of length of antennomeres I to XI (Fig. 2B) 1.0 : 0.3 : 0.5 : 0.9 : 0.9 : 0.8 : 0.9 : 0.8 : 0.8 : 0.7 : 0.7; ratio of length to width from antennomere I to XI 4.8 : 2.0 : 3.7 : 5.6 : 5.6 : 5.9 : 6.7 : 6.2 : 7.2 : 6.4 : 6.7. Gonocoxae (Fig. 2J) slender, tightly conjunct from apical 1/3 to base; each gonocoxa with eight setae from apical 1/6 to apex, apex truncate. Ventrite VIII (Fig. 2G) weakly sclerotized except apex, with several short setae at apex, and several long setae at sides, spiculum elongate. Spermathecal receptaculum (Fig. 2I) swollen; pump extremely slender and curved; sclerotized spermathecal duct slender. Bursa sclerites (Fig. 2H) elongate and apically tapering, with stout teeth along lateral margin at base; slightly curved in lateral view.

#### Food plants.

Melastomataceae: Blastus cochinchinensis Lour.; Sapindaceae: Koelreuteria henryi Dummer; Fagaceae: Castanopsis formosana (Skan) Hayata (Lee et al. 2016).

#### Distribution.

Endemic to Taiwan.

### Paleosepharia excavata

Taxon classificationAnimaliaColeopteraChrysomelidae

(Chûjô)

Figs 1D–F , 3

Monolepta excavata Chûjô, 1938 (Taiwan: Ilan, Nantou, Hualien): 144; Chûjô 1962: 114 (redescription); Chûjô 1965: 96 (Nantou); Kimoto 1969: 47 (Taichung, Nantou); Kimoto 1986: 58 (Nantou); Kimoto 1989: 254 (Taitung); Kimoto 1991: 14 (Taoyuan); Kimoto and Chu 1996: 79 (catalogue); Kimoto and Takizawa 1997: 385 (catalogue); Lee and Cheng 2010: 101 (food plants); Yang et al. 2015: 268 (catalogue).
Paleosepharia excavata : Gressitt and Kimoto 1963: 646; Beenen 2010: 485 (catalogue).
Paleosepharia polychroma Laboissière, 1938: 8 (China: Jiangsu, Jiangxi); Gressitt and Kimoto 1963: 646 (as synonym of P. excavata).

#### Type material.

*Monolepta excavata*. Lecotype ♂ (TARI), here designated, labeled: “Taiheizan [= Taipingshan, 太平山] / 23-V-1931 / Col. R. Takahashi [p, w] // CO / Type [p, y, circular label with yellow border] // *Monolepta* / *excavata* / Chûjô [h] // DET. M. CHUJO [p, w] // 2193 [p, w]”. Paralectotypes. 1♂ (TARI): “Horisha [= Puli, 埔里] / 12/V/1913 [h] / Col. M. Maki [p, w] // CO / Type [p, y, circular label with yellow border] // *Monolepta* / *excavata* / Chûjô [h] // DET. M. CHUJO [p, w] // 2194 [p, w]”; 1♂, 1♀ (TARI): “Kuaru [h] [unknown] / FORMOSA [p] / 20.VI.1937 [h] / COL. M. CHUJO [p, w] // CO / Type [p, y, circular label with yellow border] // *Monolepta* / *excavata* / Chûjô [h] // DET. M. CHUJO [p, w] // No. 1640 or 1346 [p, w]”; 1♀ (TARI): “Formosa / Musha, [= Wushe, 霧社] 1919 / V.18-VI.15. / T. Okuni [p, w] // CO / Type [p, y, circular label with yellow border] // *Monolepta* / *excavata* / Chûjô [h] // DET. M. CHUJO [p, w] // 1396 [p, w]”; 1♀ (TARI): “18/IV/1910 / Kanmon [h] [= Kuangman, 關門] / Col. I. Nitobe [p, w] // CO / Type [p, y, circular label with yellow border] // *Monolepta* / *excavata* / Chûjô [h] // DET. M. CHUJO [p, w] // 1973 [p, w]”; 1♀ (SDEI): “Formosa / Karenko [= Hualien, 花蓮], -19. / VII 20-VIII 4. / T. Okuni [p, w] // *Monolepta* / *excavata* / Chûjô [h] // DET. M. CHUJO [p, w] // 1973 [p, w] // Syntypus [p, r]”.

*Paleosepharia polychroma*. The syntypes at the Institute Royal des Sciences Naturelles de Belgique, Bruxelles, and the Naturhistoriska Riksmuseet, Stockholm were not studied. *Paleosepharia* polychroma could be a distinct species because of slight difference of color patterns on the elytra between both species. Correctly assessing the status of this species requires further study.

#### Other material examined

**(n = 72). Chiayi**: 1♂ (TARI), Alishan [阿里山], 5–9.VII.1981, leg. L. Y. Chou & S. C. Lin; **Hsinchu**: 1♂ (TARI), Kuanwu [觀霧], 6.IV.2010, leg. L.-H. Sun; 1♂ (TARI), same locality, 30.IV.2010, leg. M.-H. Tsou; 1♀ (TARI), Litungshan [李棟山], 6.VI.2010, leg. Y.-L. Lin; 1♂ (TARI), Mamei [馬美], 4.V.2008, leg. S.-F. Yu; 3♀♀ (TARI), same locality, 18.V.2008, leg. M.-H. Tsou; 1♂ (TARI), Talu trail [大鹿林道], 19.VI.2010, leg. Y.-L. Lin; 1♀ (TARI), same locality, 11.VII.2016, leg. H. Lee; **Hualien**: 1♂, 1♀ (TARI), Hsinpaiyang [新白陽], 15–20.X.2007, leg. Y.-F. Hsu; 1♀ (TARI), Huitouwan [迴頭灣], 10.VII.2007, leg. C.-F. Lee; 1♂, 1♀ (TARI), Wanjung [萬榮], 11.VI.2011, leg. M.-H. Tsou; **Ilan**: 1♀ (TARI), Chinyang [金洋], 23.X.2011, leg. C.-H. Hsieh; 1♀ (NMNS), Tsuifenghu [翠峰湖], 21.VI.1992, leg. C. Y. Li; 1♂ (NMNS), Taipingshan [太平山], 8.III.1989, leg. K. W. Huang; 1♂, 3♀♀ (TARI), same locality, 17.VIII.2007, leg. Y.-C. Chang; **Nantou**: 1♂ (NMNS), Aowanta [奧萬大], 9.IX.2008, leg. C. C. Lai; 1♂ (TARI), Fenghuangshan [鳳凰山], 10.V.2010, leg. Y.-T. Wang; 1♀ (TARI), Hoshe [和社], 23.I.2014, leg. H.-T. Yeh; 1♂ (TARI), Huakang [華岡], 13.IX.2010, leg. C.-F. Lee; 1♀ (TARI), Meifeng [梅峰], 20–22.VI.1979, leg. K. S. Lin & B. H. Chen; 1♀ (NMNS), same locality, 24.IX.1997, leg. W. T. Yang; 1♂ (NMNS), Oiwake [= Tsuifeng, 翠峰]-Tattaka [= Sungkang, 松崗], 24.VI.1961, leg. T. Shirozu; 1♂ (TARI), Tsuifeng [翠峰], 21.VI.1979, leg. K. S. Lin & B. H. Chen; 1♂ (TARI), same locality, 25–27.VI.1981, leg. K. S. Lin & W. S. Tang; 1♂ (TARI), Tungfu [同富], 8.V.2011, leg. C.-F. Lee; **Taichung**: 3♂♂, 3♀♀ (TARI), Anmashan [鞍馬山], 21.IV.2010, leg. C.-F. Lee; **Taitung**: 1♂ (TARI), Hsiangyang [向陽], 9.V.2013, leg. J.-C. Chen; 1♂ (TARI), Lichia [利嘉], 19.V.2009, leg. U. Ong; 3♀♀ (TARI), same locality, 15.VII.2014, leg. Y.-T. Chung; 1♀ (TARI), Litao [利稻], 23.VI.2010, leg. M.-H. Tsou; 4♀♀ (TARI), Liyuan [栗園], 29.III.2011, leg. C.-F. Lee; 1♀ (TARI), same locality, 24.VII.2013, leg. C.-F. Lee; 1♂ (TARI), same locality, 24.II.2014, leg. J.-C. Chen; 1♂ (TARI), same locality, 14.III.2014, leg. W.-C. Huang; 2♀♀, same locality, 28.III.2014, leg. W.-C. Huang; 1♀ (TARI), Motien [摩天], 23.VI.2010, leg. M.-H. Tsou; 1♀ (TARI), Wulu [霧鹿], 24.VI.2010, leg. M.-H. Tsou; 1♀ (TARI), same locality, 5.X.2010, leg. M.-H. Tsou; 1♂, 5♀♀ (TARI), same locality, 29.III.2011, leg. C.-F. Lee; 1♂ (NMNS), Yenping [延平], 30.VII.1992, leg. W. T. Yang; **Taoyuan**: 3♂♂, 1♀ (TARI), Hsuanyuan [萱源], 16.XII2008, leg. S.-F. Yu; 1♀ (TARI), Lalashan [拉拉山], 10.X.2009, leg. H. Lee; 2♂♂, 6♀♀ (TARI), Paling [巴陵], 8.XI.2009, leg. M.-H. Tsou; 1♀ (TARI), Tungyanshan [東眼山], 15.V.2010, leg. H. Lee; 1♂ (TARI), same locality, 5.XI.2011, leg. H. Lee.

#### Diagnosis.

Adults of *Paleosepharia excavata* are similar to those of *P. formosana* and *P. yasumatsui* in possessing two transverse black bands on yellow elytra, but this species is easily recognized by its slender, indistinctly margined transverse black bands (broad and distinctly margined bands in others). Males of these species are also similar in possessing a broader penis (less than 6.0 times longer than wide; more than 6.5 times in other species), bifurcate apex of tectum (acute apex in other species), and lacking a pair of elongate and apically curved spiculae (such spiculae present in other species). Males of *P. excavata* differ in possessing a shallow notch in the apex of the tectum (deep notch in apex of tectum in *P. yasumatsui*), recurved apex of penis (not recurved in *P. yasumatsui*), short hooked spiculae 1/2 lengths of longer spiculae (1/6 lengths of longer spiculae in *P. formosana*, 4/5 lengths of longer spiculae in *P. yasumatsui*).

#### Description.

**Figures 3.**
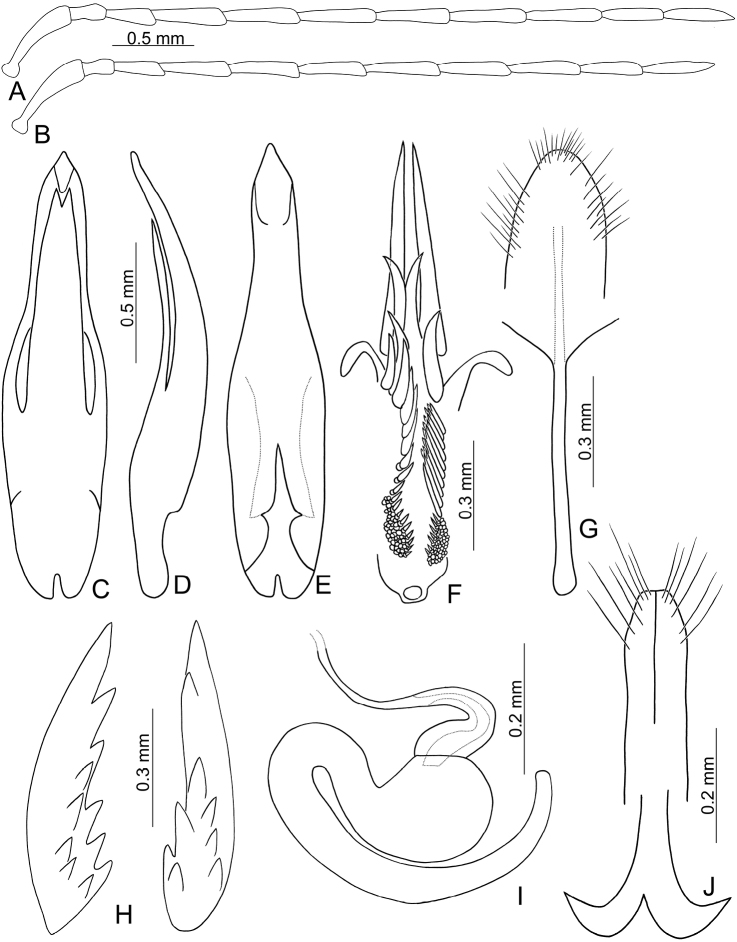
Diagnostic characters of *Paleosepharia excavata* (Chûjô). **A** Antenna, male **B** Antenna, female **C** Penis, dorsal view **D** Penis, lateral view **E** Penis, ventral view **F** Endophallic spiculae, hooked spiculae removed at right side **G** Abdominal ventrite VIII **H** Bursa sclerites, left sclerite in lateral view, right sclerite in dorsal view **I** Spermatheca **J** Gonocoxae.

Males. Length 4.8–6.1 mm, width 2.5–3.0 mm. General color reddish brown (Fig. 1D–E); antennae, scutellum, tibiae, and tarsi black; elytra yellow, with slender black stripes along each lateral margin and suture extending from base to apex, and two transverse, slender, indistinctly defined black bands at basal 1/3 and 2/3, usually abbreviated near suture and lateral margins. Antenna (Fig. 3A) filiform, ratio of length of antennomeres I to XI 1.0 : 0.4 : 0.6 : 0.9 : 0.9 : 0.9 : 0.8 : 0.8 : 0.8 : 0.7 : 0.8; ratio of length to width from antennomere I-XI 4.6 : 2.1 : 3.1 : 4.8 : 4.8 : 5.6 : 5.4 : 5.5 : 5.5 : 5.0 : 5.7. Pronotum 1.58–1.59 times wider than long; lateral margins slightly curved, basal margin slightly curved, apical margin slightly concave; disc with lateral fovea and dense minute punctures. Elytra parallel-sided; 1.49–1.56 times longer than wide; with one pair of oblique dark depressions at suture behind scutellum; disc with dense, minute punctures; apex truncate. Penis (Fig. 3C–E) elongate, 4.5 times longer than wide; parallel-sided, abruptly widened at apical 1/3, apically tapering; tectum elongate from middle to near apex, basally broadened, apex bifurcate; moderately recurved at basal 1/4 in lateral view; ventral surface with lateral areas membranous, apical membranous area short. Endophallic spiculae complex (Fig. 3F) with longest pair directed anteriorly, one pair of hooked spiculae 1/2 lengths of the longest spiculae; one pair of longitudinal rows of hooked setae starting from bases of longest spiculae, smaller near base; one row of elongate, apically tapering setae covering surface behind hook-like setae; and one paired cluster of short, hooked setae near base.

Females. Length 5.4–6.3 mm, width 2.8–3.3 mm. Similar to male (Fig. 1F); but elytra lacking oblique depressions, anterior transverse black band extending to suture; ratio of length of antennomeres I to XI (Fig. 3B) 1.0 : 0.3 : 0.6 : 0.8 : 0.7 : 0.7 : 0.8 : 0.8 : 0.8 : 0.7 : 0.8; ratio of length to width from antennomere I to XI 4.1 : 1.9 : 3.3 : 4.5 : 4.4 : 4.4 : 5.1 : 4.9 : 5.5 : 4.8 : 6.0. Gonocoxae (Fig. 3J) slender, tightly conjunct from apical 1/3 to base; each gonocoxa with six or seven setae from apical 1/6 to apex, apex truncate. Ventrite VIII (Fig. 3G) weakly sclerotized medially, with several short setae at apex, and two rows of long setae at sides, spiculum extremely elongate. Spermathecal receptaculum (Fig. 3I) swollen; pump extremely slender and curved; sclerotized spermathecal duct slender. Bursa sclerites (Fig. 3H) elongate and apically tapering, with stout teeth along lateral margin at base; slightly curved in lateral view.

#### Food plant.

Betulaceae: Alnus formosana (Burkill ex Forbes & Hemsl.) Makino (Lee and Cheng 2010).

#### Distribution.

Taiwan, China.

### Paleosepharia formosana

Taxon classificationAnimaliaColeopteraChrysomelidae

(Chûjô) comb. n.

Figs 4A–C , 5

Monolepta formosana Chûjô, 1935: 172 (Taipei, Nantou); Chûjô 1938: 143 (Chiayi); Chûjô 1962: 135 (redescription; Taitung); Chûjô 1965: 97 (Taipei, Nantou); Kimoto 1965: 488 (Nantou); Kimoto 1966: 32 (Chiayi, Nantou); Kimoto 1969: 48 (Taipei, Taichung, Nantou); Kimoto 1986: 58 (Nantou); Kimoto 1989: 254 (Taipei, Kaohsiung); Kimoto and Chu 1996: 79 (catalogue); Kimoto and Takizawa 1997: 385 (catalogue); Lee and Cheng 2007: 112 (food plants); Beenen 2010: 483 (catalogue); Yang et al. 2015: 268 (catalogue).

#### Type material.

Lectotype ♂ (TARI), here designated, labeled: “Sôzan [h] [= Yangmingshan, 陽明山] / FORMOSA [p] / 24.IX.1932 [h] / COL. M. CHUJO [P] // CO / Type [p, y, circular label with yellow border] // *Monolepta* / *formosana* / Chûjô [h] // DET. M. CHUJO [p, w] // No. 1341 [p, w]”. Paralectotypes. 3♂♂, 1♀ (TARI), same data as lectotype, but with “1342, 1343, 1901, or 1902; 1♂ (SDEI): “Fuhosho [= Wucheng, 五城] / Formosa / Sauter [p] VIII [h] 07–09 [p, w] // *Monolepta* / *formosana* / Chûjô [h] // DET. M. CHUJO [p, w] // Syntypus [p, r]”.

#### Other material examined

**(n = 90). Hsinchu**: 1♀ (TARI), Litungshan [李棟山], 6.VI.2010, leg. Y.-L. Lin; 2♂♂ (TARI), Lupi [魯壁], 26.VII.2008, leg. M.-H. Tsou; **Hualien**: 1♂ (TARI), Huitouwan [迴頭灣], 10.VII.2007, leg. C.-F. Lee; 1♀ (TARI), Huojanting [豁然亭], 7–14.IV.2007, leg. Y.-F. Hsu; 1♀ (TARI), Wanjung [萬榮], 11.VI.2011, leg. M.-H. Tsou; **Ilan**: 1♂, 1♀ (NMNS), Fushan Botanical Garden [福山植物園], 26–27.VIII.2003, leg. M. L. Chan; 2♀♀ (TARI), same locality, 2.IV.2008, leg. M.-H. Tsou; 2♀♀ (TARI), same locality, 8.V.2008, leg. S.-F. Yu; 1♀ (TARI), same locality, 8.VII.2013, leg. Y.-T. Wang; 1♀ (TARI), Yingtzuling [鶯仔嶺], 7.IV.2012, leg. Y.-L. Lin; **Kaoshiung**: 1♂ (TARI), Tengchih [藤枝], 7.IX.2012, leg. W.-C. Liao; 1♀ (TARI), Tona trail [多納林道], 3.II.2013, leg. B.-X. Guo; **Nantou**: 1♂ (NMNS), Huisun Forest [惠蓀林場], 12–13.XI.1998, leg. C. S. Lin & W. T. Yang; 1♂ (TARI), 17.XI.2008, same locality, leg. M.-H. Tsou; 1♀ (NMNS), Lake Candidius [= Jihyuehtan, 日月潭], 26.VI.1961, leg. T. Shirozu; 3♀♀ (TARI), Lienhuachih [蓮華池], 23–26.V.1980, leg. K. S. Lin & B. H. Chen; 1♀ (TARI), same locality, 26.VII.1984, leg. K. S. Lin; 1♀ (NMNS), same locality, 12.VII.-9.VIII.2004, leg. C. S. Lin & W. T. Yang; 1♂ (NMNS), same locality, 4.X.-15.XI.2004, leg. C. S. Lin & W. T. Yang; 1♂ (TARI), same locality, 22.III.2009, leg. U. Ong; 3♂♂, 3♀♀ (TARI), Lushan [盧山], 27–31.V.1980, leg. K. S. Lin & L. Y. Chou; 1♂ (TARI), same locality, 28.VI.1981, leg. K. S. Lin & W. S. Tang; 1♂ (NMNS), Nanshanchi [南山溪], 21.VI.1965, leg. B. S. Chang; 1♂ (TARI), same locality, 11.VII.2007, leg. M.-H. Tsou; 1♀ (TARI), Sungchuankang [松泉崗], 25.VII.2010, leg. T.-Y. Liu; 1♂ (TARI), Sunglintsun [松林村], 9.VII.2007, leg. M.-H. Tsou; 2♂♂ (TARI), Tungpu [東埔], 25–29.IX.1980, leg. L. Y. Chou & T. Lin; 1♀ (TARI), same locality, 28.IV.-2.V.1981, leg. T. Lin & C. J. Lee; 1♂, 1♀ (TARI), same locality, 5–8.X.1981, leg. T. Lin & W. S. Tang; 2♂♂, 2♀♀ (TARI), same locality, 19–23.VII.1982, leg. L. Y. Chou & T. Lin; 2♂♂ (TARI), same locality, 22–25.XI.1982, leg. K. C. Chou & S. P. Huang; 1♀ (TARI), same locality, 20–24.VI.1983, leg. K. C. Chou & C. Y. Wong; 2♂♂, 4♀♀ (TARI), same locality, 23–27.VII.1984, K. C. Chou & C. H. Yang; 1♀ (TARI), Wanfengtsun [萬豐村], 4.X.2007, leg. W.-T. Liu; 3♂♂, 1♀ (TARI), same locality, 8.VII.2008, leg. W.-T. Liu; 1♂ (TARI), Wushe [霧社], 4.VIII.1981, leg. T. Lin & W. S. Tang; **Pingtung**: 2♀♀ (TARI), Ali [阿禮], 17.II.2016, leg. Y.-T. Chung; 1♀ (TARI), Lilungshan [里龍山], 17.VIII.2014, leg. Y.-T. Chung; 1♀ (TARI), Kenting [墾丁], 5–9.XII.1982, leg. S. C. Lin & S. P. Huang; 1♀ (TARI), Nanjenhu [南仁湖], 15.III.2010, leg. M.-H. Tsou; 1♂ (TARI), Tahanshan [大漢山], 20.VII.2007, leg. S.-F. Yu; 1♂ (TARI), same locality, 6.II.2008, leg. M.-H. Tsou; 2♂♂ (TARI), 13–14.VIII.2011, leg. Y.-T. Wang; 1♀ (TARI), same locality, 20.X.2012, leg. W.-C. Liao; 1♂ (TARI), same locality, 15.XII.2012, leg. W.-C. Liao; 1♂ (TARI), same locality, 10.VII.2013, leg. Y.-T. Chung; 1♀ (TARI), 10.IV.2017, leg. Y.-T. Chung; 3♀♀ (TARI), Wutai [霧台], 15–16.III.2009, leg. Y.-F. Hsu; 1♀ (TARI), same locality, 12.IV.2009, U. Ong; **Taipei**: 1♂, 1♀ (NMNS), Fushan [福山], 28–29.V.2004, leg. C. S. Lin & W. T. Yang; 1♂, 1♀ (NMNS), Rimogan [= Fushan, 福山]-Magan, 10.VII.1961, leg. T. Shirozu; 1♀ (TARI), Wulai [烏來], 4.III.2007, leg. S.-F. Yu; 1♂ (TARI), same locality, 30.III.2007, leg. C.-F. Lee; **Taichung**: 1♂, 1♀ (NMNS), Bojinjiashan [波津加山], 8.X.1987, leg. I. C. Hsu; 2♂♂, 1♀ (TARI), Chiapaotai [佳保台], 14–18.X.1980, leg. K. S. Lin & C. H. Wang; 1♀ (NMNS), Kukuan [谷關], 11–12.IV.1986, leg. C. S. Lin; **Taitung**: 1♂ (TARI), Lichia [利嘉], 24.IV.2008, leg. C.-L. Hsiao; 1♀ (TARI), Liyuan [栗園], 29.III.2011, leg. C.-F. Lee; 1♂ (TARI), Shouka [壽卡], 13.VI.2013, leg. Y.-T. Chung; 2♂♂ (TARI), same locality, 27.X.2013, leg. W.-C. Liao; 1♀ (TARI), same locality, 19.IV.2015, leg. W.-C. Liao; 1♂, 1♀ (TARI), Tulanshan [都蘭山], 14.IX.2014, leg. Y.-T. Chung; 1♂ (TARI), Wulu [霧鹿], 5.X.2010, leg. M.-H. Tsou; **Taoyuan**: 1♀ (TARI), Hsiaowulai [小烏來], 29.IX.2009, leg. M.-H. Tsou; 1♂, 2♀♀ (TARI), Paling [巴陵], 3–5.V.1983, leg. K. C. Chou & C. C. Pan; 1♂ (TARI), Tungyanshan [東眼山], 12.IV.2007, leg. M.-H. Tsou; 1♂ (TARI), same locality, 20.IX.2007, leg. S.-F. Yu.

#### Diagnosis.

Adults of *Paleosepharia* formosana are similar to those of *P. yasumatsui* in possessing two broad, well-defined transverse black bands on the elytra. However, males of *P. yasumatsui* are easily separated from those of *P. formosana* by the absence of anterior transverse black bands near the suture (present in *P. formosana*), and presence of clusters of stout setae behind the scutellum (lacking stout setae in *P. formosana*). Females of *P. yasumatsui* are similar to those of *P. formosana* but differ by their straight lateral margins of the elytra and continuously arcuate posterior black stripes (rounded elytra and independently arcuate black stripes in *P. formosana*). Males of *P. formosana*, *P. excavata*, and *P. yasumatsui* are similar in possessing a broad penis (less than 6.0 times longer than wide; more than 6.5 times in other species), bifurcate apex of tectum (acute apex in other species), lacking a pair of elongate, apically curved spiculae (such spiculae present in other species). They differ in possessing a shallow notch of the apex of the tectum (deep notch in *P. yasumatsui*), recurved apex of the penis (not recurved in *P. yasumatsui*), short hooked spiculae 1/6 the lengths of longer spiculae (1/2 length of longer spiculae in *P. excavata*, 4/5 length of longer spiculae in *P. yasumatsui*).

#### Description.

**Figures 4.**
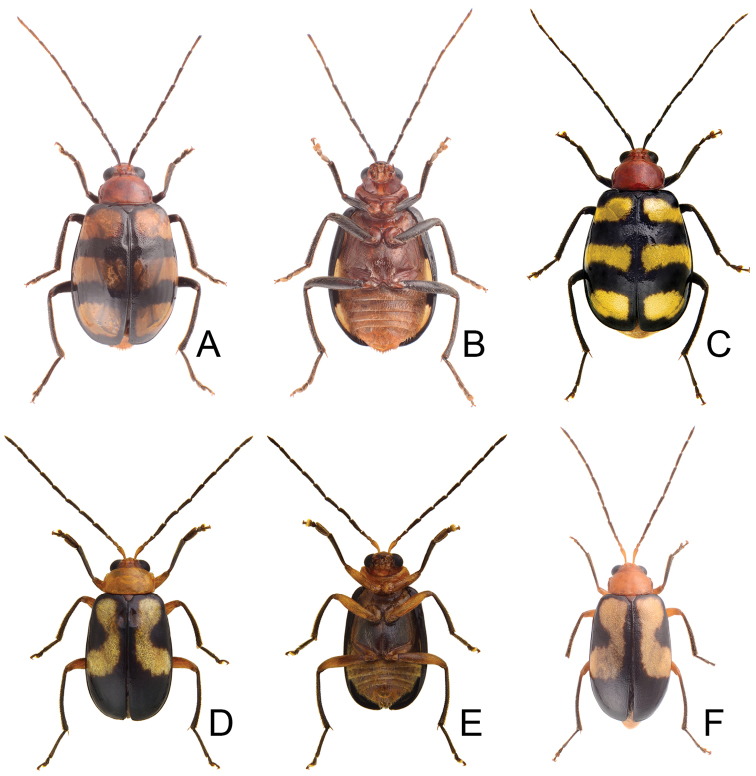
Habitus of *Paleosepharia* species. **A** *P. formosana* (Chûjô), male, dorsal view **B** Ditto, ventral view **C** *P. formosana* (Chûjô), female, color variation, dorsal view **D** *P. nantouensis* (Kimoto), male, dorsal view **E** Ditto, ventral view **F** nantouensis (Kimoto), female, dorsal view.

**Figures 5.**
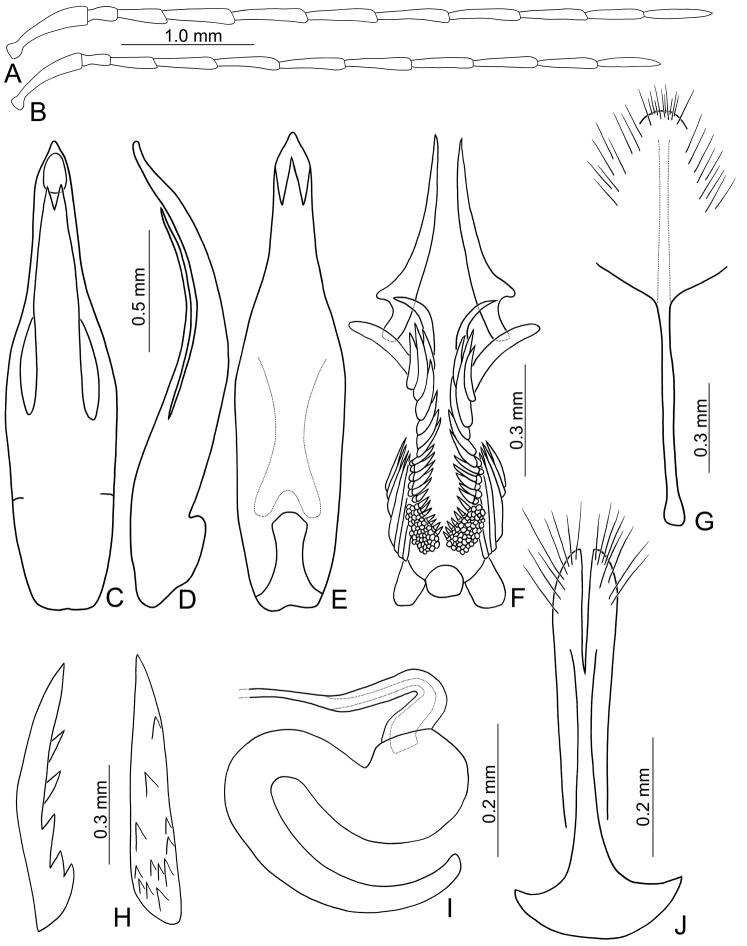
Diagnostic characters of *Paleosepharia formosana* (Chûjô). **A** Antenna, male **B** Antenna, female **C** Penis, dorsal view **D** Penis, lateral view **E** Penis, ventral view **F** Endophallic spiculae **G** Abdominal ventrite VIII **H** Bursa sclerites, left sclerite in lateral view, right sclerite in dorsal view **I** Spermatheca **J** Gonocoxae.

Males. Length 5.6–6.3 mm, width 3.2–3.5 mm. General color reddish brown (Fig. 4A–B); antennae and legs black; scutellum reddish brown; elytra yellow, with wide black stripe along lateral margins and suture, extending from base to apex, and two transverse, wide black bands at basal 1/3 and 2/3. Antenna (Fig. 5A) filiform, ratio of length of antennomeres I to XI 1.0 : 0.3 : 0.6 : 0.8 : 0.9 : 0.8 : 0.8 : 0.8 : 0.8 : 0.7 : 0.8; ratio of length to width from antennomere I to XI 4.4 : 2.0 : 3.2 : 4.7 : 5.3 : 5.7 : 5.7 : 6.5 : 6.2 : 5.8 : 6.8. Pronotum 1.76–1.78 times wider than long; lateral margins straight; basal margin slightly curved, apical margin slightly concave; disc evenly convex, with reticulate microsculpture and dense, minute punctures. Elytra widest at apical 1/3; 1.34–1.35 times longer than wide; with one pair of longitudinal depressions near suture, behind scutellum; disc with dense, minute punctures; apex truncate. Penis (Fig. 5C–E) elongate, 4.4 times longer than wide; parallel-sided, abruptly widened at apical 1/3, apically tapering; tectum elongate from middle to near apex, parallel-sided; apex bifurcate; moderately recurved at basal 1/4 in lateral view; ventral surface with lateral areas membranous, apical membranous area short and with tapering process from lower part of membranous area. Endophallic spiculae (Fig. 5F) with longest pair directed anteriorly, another pair of shorter, hooked spiculae curved outside near apex; one pair of longitudinal rows of hooked setae originating from bases of longest setae and reaching near base, smaller near base; one paired cluster of short hooked setae covering surface near base; one row of long, apically tapering setae on side, smaller near apex.

Females. Length 5.7–6.7 mm, width 3.4–3.6 mm. Similar to male (Fig. 4C); but elytra lacking longitudinal depressions; ratio of length of antennomeres I to XI (Fig. 5B) 1.0 : 0.3 : 0.6 : 0.8 : 0.7 : 0.8 : 0.8 : 0.8 : 0.8 : 0.7 : 0.8; ratio of length to width from antennomere I to XI 5.3 : 2.4 : 4.1 : 5.2 : 4.9 : 5.3 : 5.3 : 5.8 : 5.9 : 5.5 : 6.5. Gonocoxae (Fig. 5J) slender, tightly conjunct from apical 1/3 to base; each gonocoxa with eight setae from apical 1/6 to apex, apex curved. Ventrite VIII (Fig. 5G) weakly sclerotized apically, apical margin curved, with several short setae and several long setae at sides, spiculum extremely elongate. Spermathecal receptaculum (Fig. 5I) swollen; pump extremely slender and curved; sclerotized spermathecal duct slender. Bursa sclerites (Fig. 5H) elongate and apically tapering, with dense, stout teeth near base; slightly curved in lateral view.

#### Food plants.

Saxifragaceae: Itea parviflora Hemsl.; Hamamelidaceae: Liquidambar formosana Hance; Myrsinaceae: Ardisia sieboldii Miq (Lee and Cheng 2007).

#### Remarks.

Lee and Cheng (2007) misidentified males of *Paleosepharia* formosana; they are re-identified as belonging to females of *P. yasumatsui*.

#### Distribution.

Endemic to Taiwan.

### Paleosepharia nantouensis

Taxon classificationAnimaliaColeopteraChrysomelidae

(Kimoto) comb. n.

Figs 4D–F , 6

Monolepta nantouensis Kimoto, 1996: 38; Kimoto and Takizawa 1997: 386 (catalogue); Beenen 2010: 484 (catalogue); Lee and Cheng 2010: 102 (food plant); Yang et al. 2015: 270 (catalogue).

#### Type material.

Paratype 1♀ (KMNH): “Baibara [h, w] // C. TAIWAN, / End of 1950’s [p, w] // *Monolepta* / *nantouensis* / Kimoto, n. sp. [h] / Det. S. Kimoto. 19 [p, w] // PHOTO [p, r] // PARATYPE [p, b]”.

#### Other material examined

**(n = 22). Hsinchu**: 2♀♀ (TARI), Chenhsipao [鎮西堡], 26.VII.2014, leg. Y.-L. Lin; 4♂♂ (TARI), Chienshih [尖石], 12.VII.2009, leg. M.-H. Tsou; 1♀ (TARI), Lupi [魯壁], 20.VII.2008, leg. M.-H. Tsou; 1♀ (TARI), same locality, 26.VII.2008, leg. M.-H. Tsou; **Ilan**: 1♀ (TARI), Suyuan [思源], 23.VII.2008, leg. H.-Y. Lee; 1♀ (TARI), same locality, 8.VIII.2014, leg. S.-F. Yu; **Kaoshiung**: 1♂ (TARI), Meilungshan [美瓏山], 15.VI.2016, leg. B.-X. Guo; Taichung: 1♂ (NMNS), Anmashan [鞍馬山], 18.VII.2005, leg. Y.-L. Chen; **Taitung**: 2♂♂, 3♀♀ (TARI), Liyuan [栗園], 24.VII.2013, leg. C.-F. Lee; **Taoyuan**: 1♂, 1♀ (TARI), Lalashan [拉拉山], 2.VIII.2008, leg. M.-H. Tsou; 1♂ (TARI), same locality, 7.VIII.2008, leg. H.-J. Chen; 1♀ (TARI), same locality, 30.VIII.2008, leg. M.-H. Tsou; 1♀ (NMNS), Shanpaling [上巴陵], 26.VIII.1987, leg. I. C. Hsu; 1♂ (TARI), Tamanshan [塔曼山], 3.VIII.2008, leg. M.-H. Tsou; 1♀ (TARI), same but with “leg. S.-F. Yu”.

#### Description.

**Figures 6.**
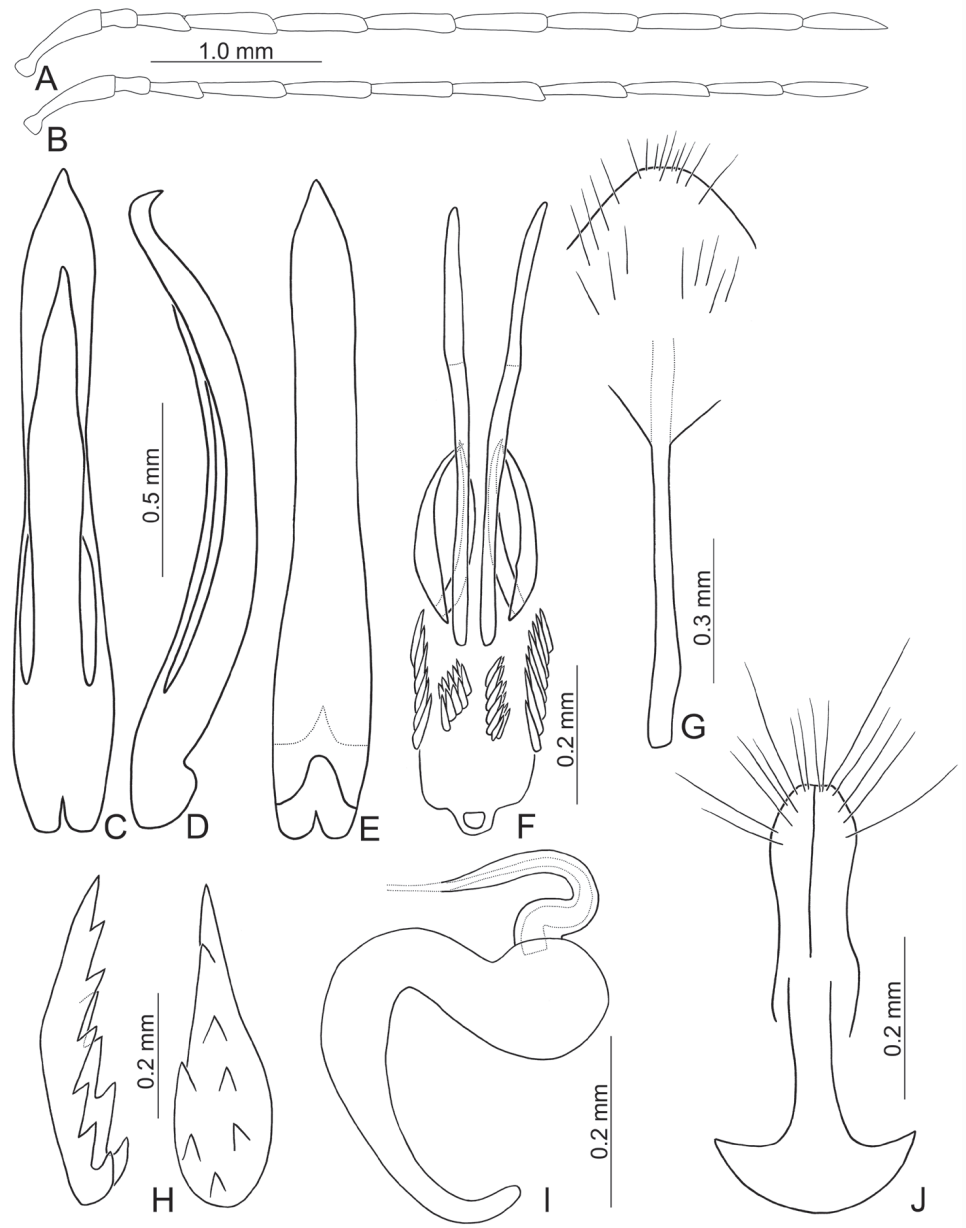
Diagnostic characters of *Paleosepharia nantouensis* (Kimoto). **A** Antenna, male **B** Antenna, female **C** Penis, dorsal view **D** Penis, lateral view **E** Penis, ventral view **F** Endophallic spiculae **G** Abdominal ventrite VIII **H** Bursa sclerites, left sclerite in lateral view, right sclerite in dorsal view **I** Spermatheca **J** Gonocoxae.

Males. Length 4.7–5.2 mm, width 2.4–2.6 mm. General color yellowish brown (Fig. 4D–E); antennae, except two basal antennomeres, tibiae, tarsi, and metaventrite blackish brown; scutellum yellowish brown; elytra black, with one pair of broad, curved white stripes from near base to middle, curved inwards at middle. Antenna (Fig. 6A) filiform, ratio of length of antennomeres I to XI 1.0 : 0.3 : 0.5 : 0.9 : 0.9 : 0.9 : 0.9 : 0.9 : 0.9 : 0.8 : 0.9; ratio of length to width from antennomere I to XI 3.7 : 2.0 : 2.9 : 5.1 : 5.1 : 4.9 : 5.0 : 4.9 : 5.2 : 4.9 : 5.6. Pronotum 1.69–1.81 times wider than long; lateral margins slightly curved, basal margin slightly curved, apical margin slightly concave; disc evenly convex, with reticulate microsculpture and dense, minute punctures and one pair of weak fovea at sides. Elytra parallel-sided; 1.51–1.57 times longer than wide; with one pair of depressions near suture in basal 1/3; disc with dense, minute punctures; apex truncate. First tarsomeres of front legs dorso-ventrally flattened. Penis (Fig. 6C–E) extremely slender, 6.8 times longer than wide; parallel-sided, posterior widened at middle, apically tapering; tectum elongate from basal 1/5 to apical 1/6, widest at middle and as wide as penis, apically tapering, apex acute; moderately recurved at middle and near apex in lateral view; ventral surface without membranous areas. Endophallic spiculae complex (Fig. 6F) with the longest pair apically curved; also possessing two pairs of long hooked spiculae of equal length, one pair curved inwards, the other curved outwards; a pair of longitudinal rows of hook-like setae distal to bases of longest spiculae; one longitudinal pair of long, apically tapering setae along sides distal to bases of longest spiculae.

Females. Length 5.3–6.3 mm, width 2.6–3.3 mm. Similar to males (Fig. 4F); but elytra lacking oblique depressions; first tarsomeres of front legs normal; ratio of length of antennomeres I to XI (Fig. 6B) 1.0 : 0.3 : 0.5 : 0.9 : 0.9 : 0.8 : 0.9 : 0.8 : 0.8 : 0.7 : 0.7; ratio of length to width from antennomere I-XI 4.8 : 2.0 : 3.7 : 5.6 : 5.6 : 5.9 : 6.7 : 6.2 : 7.2 : 6.4 : 6.7. Gonocoxae (Fig. 6J) slender, tightly conjunct from apical 1/3 to base; each gonocoxa with eight setae from apical 1/6 to apex, apex truncate. Ventrite VIII (Fig. 6G) weakly sclerotized except apex, with several short setae at apex, and several long setae at sides, spiculum elongate. Spermathecal receptaculum (Fig. 6I) swollen; pump extremely slender and curved; sclerotized spermathecal duct slender. Bursa sclerites (Fig. 6H) elongate and apically tapering, with stout teeth along lateral margin at base; slightly curved in lateral view.

#### Food plant.

Fagaceae: Castanopsis carlesii (Hemsl.) Hayata (Lee and Cheng 2010).

#### Distribution.

Endemic to Taiwan.

### Paleosepharia yasumatsui

Taxon classificationAnimaliaColeopteraChrysomelidae

(Kimoto) comb. n.

Figs 7 , 8

Monolepta yasumatsui Kimoto, 1969: 51 (Nantou, Ilan); Kimoto 1991: 15 (Kaohsiung); Kimoto and Chu 1996: 83 (catalogue); Kimoto and Takizawa 1997: 387 (catalogue); Beenen 2010: 485 (catalogue); Lee and Cheng 2010: 103 (food plants); Yang et al. 2015: 274 (catalogue).

#### Type material.

Holotype ♂ (KUEC), labeled: “(Taiwan) / Nanshanchi [南山溪] / Nantou Hsien [p, w] // 30[h].vi.1965 / S. Kimoto [p, w] // Japan-U. S. / Co-op. Sic. / Programme [p, y] // *Monolepta* / *yasumatsui* / Kimoto, n. sp. [h, w] // HOLOTYPE [p, r]”. Paratypes. 1♂ (KMNH): “(TAIWAN) / Penpuchi [本部溪] / Nontou Hsien / 13. VII. 1966 / H. Kamiya leg. [p, w] // *Monolepta* / *yasumatsui* / Kimoto, n. sp. [h, w] // PARATYPE [p, b]”; 1♂ (KMNH): “(Taiwan) [p] / (Taipinshan [太平山]) / Ilan [h] Hsien [p, w] // 5.viii.1967 [h] / B. S. Chang [p, w] // *Monolepta* / *yasumatsui* / Kimoto, n. sp. [h, w] // PARATYPE [p, b]”.

#### Other material examined

**(n = 68). Hsinchu**: 1♂, 1♀ (TARI), Mamei [馬美], 18.V.2008, leg. S.-F. Yu; 1♀ (TARI), Talu trail [大鹿林道], 17.III.2012, leg. Y.-L. Lin; **Hualien**: 1♂ (TARI), Wanjung [萬榮], 11.VI.2011, leg. M.-H. Tsou; **Ilan**: 2♀♀ (TARI), Fushan Botanical Park [福山植物園], 1.IV.2008, leg. M.-H. Tsou; 1♀ (TARI), same but with “leg. H.-J. Chen; 1♂ (TARI), same locality, 13.IV.2011, leg. C.-F. Lee; 1♂ (TARI), same locality, 3.VII.2013, leg. Y.-T. Wang; 1♂, 1♀ (NMNS), Mingchi [明池], 18.II.2000, leg. M. L. Chan; 1♂ (TARI), 17.III.2007, leg. M.-H. Tsou; 2♂♂ (TARI), same locality, 16.VIII.2008, leg. M.-H. Tsou; 1♂ (TARI), same locality, 5.IV.2009, leg. M.-H. Tsou; 1♂ (TARI), Shenmihu [神秘湖], 17.IV.2010, leg. M.-H. Tsou; 1♂ (TARI), Taipingshan [太平山], 15.XI.2007, leg. S.-S. Li; 2♀♀ (TARI), Tungshan [銅山], 6.IX.2010, leg. Y.-F. Hsu; **Kaoshiung**: 1♂ (TARI), Chungchihkuan [中之關], 10–13.X.2012, leg. L.-P. Hsu; 1♂ (TARI), Tengchih [藤枝], 27.IX.2013, leg. W.-C. Liao; 2♀♀ (TARI), same locality, 30.VIII.2014, leg. B.-X. Guo; 1♂, 1♀ (TARI), same locality, 2.IV.2016, leg. W.-C. Liao; 1♀ (TARI), Tianchih [天池], 11.X.2012, leg. L.-P. Hsu; 1♂ (NMNS), Tona trail [多納林道], 30.IV.1998, leg. M. L Chan; 1♀ (TARI), 20.III.2010, leg. U. Ong; **Nantou**: 1♂ (TARI), Huisunlinchang [惠蓀林場], 26.IV.2014, leg. B.-X. Guo; 1♂ (TARI), same locality, 23.IV.2015, leg. Y.-T. Chung; 2♀♀ (NMNS), Lienhuachih [蓮華池], 21.IV.1998, leg. M. M. Yang & H. T. Chan; 1♂ (TARI), Nanshanchi [南山溪], 25.VII.2008, leg. W.-T. Liu; 1♀ (TARI), same locality, 7.IV.2010, leg. Y.-T. Wang; 1♂ (NMNS), Peitungyenshan [北東眼山], 23–25.IX.1998, leg. W. T. Yang; 1♂ (TARI), Pihu [碧湖], 27.IX.2011, leg. J.-C. Chen; **Pingtung**: 2♂♂ (TARI), Jinshuiying [浸水營], 19.XI.2009, leg. J.-C. Chen; 1♂ (TARI), same locality, 12.IX.2011, leg. J.-C. Chen; 1♂ (TARI), same locality, 12.IV.2012, leg. C.-F. Lee; 1♀ (TARI), same locality, 27.IV.2014, leg. J.-C. Chen; 1♂ (TARI), Tahanshan [大漢山], 6.VIII.2013, leg. Y.-T. Chung; 1♂ (TARI), Wutai [霧台], 12.IV.2009, leg. U. Ong; 2♀♀ (TARI), same locality, 17.V.2009, leg. U. Ong; **Taipei**: 1♀ (TARI), Wulai [烏來], 4.III.2007, leg. S.-F. Yu; 1♂ (TARI), same locality, 17.V.2008, leg. M.-H. Tsou; **Taichung**: 1♂ (TARI), Anmashan [鞍馬山], 22.III.2011, leg. C.-F. Lee; 1♂ (TARI), same locality, 19.X.2011, leg. C.-F. Lee; 1♂ (NMNS), Chiapaotai [佳保台], 6.XII.1991, leg. Y. C. Shiau; 1♀ (TARI), Kukuan [谷關], 19.III.2014, leg. C.-F. Lee; Tainan: 1♀ (TARI), Kantoushan [崁頭山], 20.X.2013, leg. W.-C. Liao; **Taitung**: 3♀♀ (TARI), Lichia [利嘉], 9.IV.2016, S.-P. Wu; 2♂♂, 1♀ (TARI), Motien [摩天], 5.X.2010, leg. C.-F. Lee; 1♂, 1♀ (TARI), Wulu [霧鹿], 5.X.2010, leg. M.-H. Tsou; **Taoyuan**: 1♂ (TARI), Fuhsing [復興], 12.IV.2009, leg. M.-H. Tsou; 2♂♂ (TARI), Hsiaowulai [小烏來], 29.IX.2009, leg. M.-H. Tsou; 8♂♂ (TARI), same locality, 10.X.2009, leg. M.-H. Tsou; 1♂ (TARI), Lalashan [拉拉山], 26.X.2008, leg. M.-H. Tsou; 1♂ (TARI), Paling [巴陵], 8.XI.2009, leg. M.-H. Tsou; 1♂ (TARI), Sankuang [三光], 17.X.2009, leg. Y.-L. Lin; 2♂♂ (NMNS), Shanpaling [上巴陵], 26.VIII.1987, leg. I. C. Hsu; 1♀ (TARI), Tungyanshan [東眼山], 20.IX.2007, leg. S.-F. Yu.

#### Diagnosis.

Adults of *P. yasumatsui* are similar to those of *P. formosana* in possessing two broad, distinctly margined transverse black bands on the elytra. However, males of *P. yasumatsui* are easily separated from those of *P. formosana* by the absence of an anterior, transverse black band near the suture (present in *P. formosana*), and presence of clusters of stout setae behind the scutellum (lacking stout setae in *P. formosana*). Females of *P. yasumatsui* are similar to those of *P. formosana* but differ in the straight lateral margin of the elytra and continuously arcuate, posterior black stripes (rounded elytra and separated arcuate black stripes in *P. formosana*). Males of *P. formosana*, *P. excavata*, and *P. yasumatsui* are similar in possessing a broader penis (less than 6.0 times longer than wide; more than 6.5 times in other species), bifurcate apex of tectum (acute apex in other species), and lacking a pair of elongate, apically curved spiculae (such spiculae present in other species). Males of *P. yasumatsui* differ in having a deep notch at the apex of the tectum (shallow notch at apex of tectum in other species), curved apex of penis (recurved in other species), short, hooked spiculae 4/5 sizes of long spiculae (1/2 sizes of long spiculae in *P. excavata*, 1/6 sizes of long spiculae in *P. formosana*).

#### Description.

**Figures 7.**
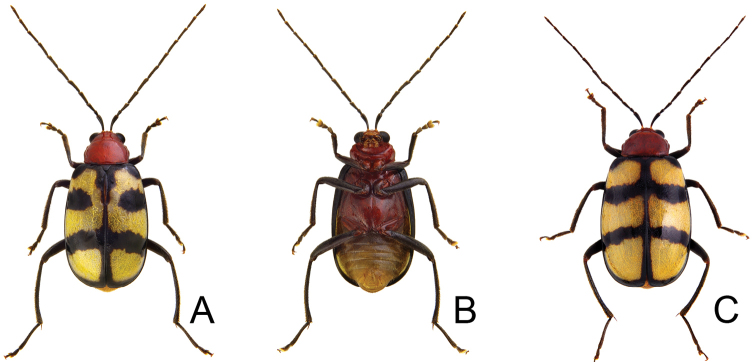
Habitus of *Paleosepharia yasumatsui* (Kimoto). **A** Male, dorsal view **B** Ditto, ventral view **C** Female, dorsal view.

**Figures 8.**
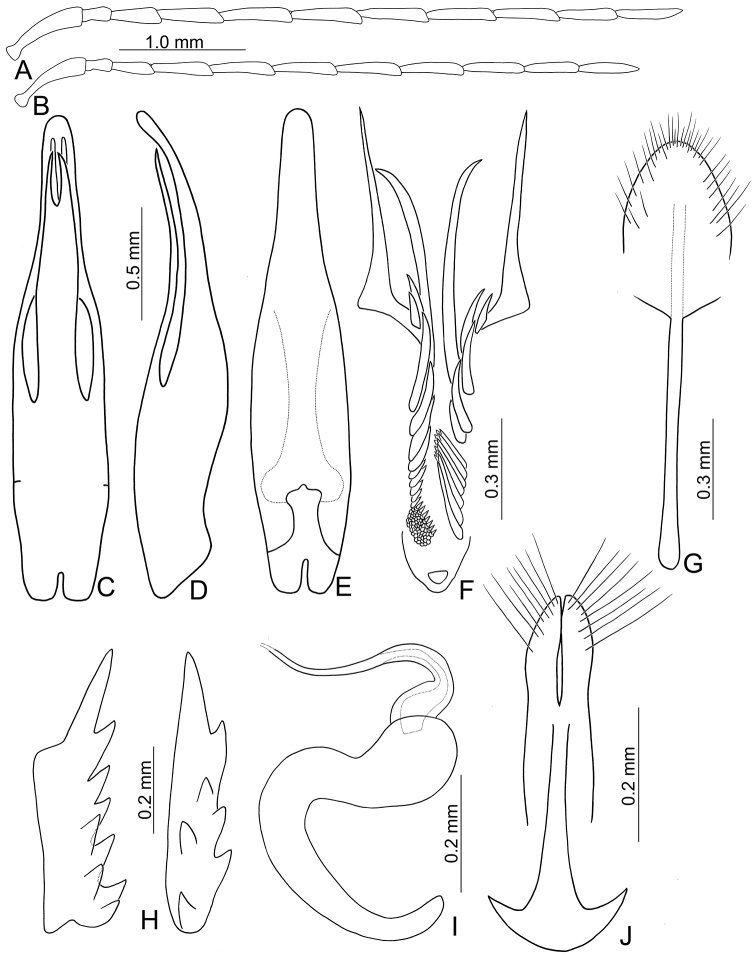
Diagnostic characters of *Paleosepharia yasumatsui* (Kimoto). **A** Antenna, male **B** Antenna, female **C** Penis, dorsal view **D** Penis, lateral view **E** Penis, ventral view **F** Endophallic spiculae, hooked spiculae removed at right side **G** Abdominal ventrite VIII **H** Bursa sclerites, left sclerite in lateral view, right sclerite in dorsal view **I** Spermatheca **J** Gonocoxae.

Males. Length 5.2–6.0 mm, width 2.7–3.3 mm. General color reddish brown (Fig. 7A–B); antennae and legs black; scutellum reddish brown; elytra yellow, with wide black stripe along lateral margins and suture extending from base to apex; two transverse wide black bands at basal 1/3 and 2/3, anterior band abbreviated near suture. Antenna (Fig. 8A) filiform, ratio of length of antennomeres I to XI 1.0 : 0.3 : 0.6 : 0.8 : 0.9 : 0.9 : 0.9 : 0.8 : 0.8 : 0.7 : 0.8; ratio of length to width from antennomere I-XI 4.2 : 2.0 : 3.2 : 4.2 : 5.1 : 5.1 : 5.1 : 5.5 : 5.4 : 5.1 : 6.7. Pronotum 1.44–1.51 times wider than long; lateral margins slightly curved, basal margin slightly curved, apical margin slightly concave; disc evenly convex, with reticulate microsculpture and dense, minute punctures, and with one pair weak fovea at sides. Elytra subparallel, gradually broadened towards apices; 1.45–1.49 times longer than wide; with one pair of weak depressions near suture at basal 1/3; with paired clusters of long setae behind scutellum; disc with dense, minute punctures; apex truncate. Penis (Fig. 8C–E) elongate, 5.5 times longer than wide; parallel-sided, strongly widened at apical 1/3, apically curved; tectum elongate from middle to near apex, parallel-sided; apex deeply bifurcate; slightly recurved at basal 1/4 in lateral view; ventral surface with lateral areas membranous. Endophallic spiculae complex (Fig. 8F) with one longest pair directed anteriorly, another shorter pair of hooked spiculae; one pair of elongate rows of hooked setae distal to base of longest spiculae, smaller near base; one paired cluster of short and apically tapering setae near base; one pair of elongate rows of long, apically tapering setae covered by hooked setae.

Females. Length 4.6–5.7 mm, width 2.6–3.1 mm. Similar to male (Fig. 7C); but elytra lacking depressions and clusters of long setae, and anterior black band extending to suture, ratio of length of antennomeres I to XI (Fig. 8B) 1.0 : 0.3 : 0.5 : 0.9 : 0.9 : 0.8 : 0.9 : 0.9 : 0.8 : 0.7 : 0.9; ratio of length to width from antennomere I to XI 4.7 : 2.1 : 3.4 : 5.4 : 5.3 : 5.3 : 6.1 : 5.6 : 6.3 : 5.4 : 6.0. Gonocoxae (Fig. 8J) slender, tightly conjunct from apical 1/3 to base; each gonocoxa with seven to eight setae from apical 1/6 to apex, apex truncate. Ventrite VIII (Fig. 8G) medially and weakly sclerotized with apical margin curved, with dense, short setae at apex, and several long setae at sides, spiculum extremely elongate. Spermathecal receptaculum (Fig. 8I) swollen; pump extremely slender and curved; sclerotized spermathecal duct slender. Bursa sclerites (Fig. 8H) elongate and apically tapering, two elongate rows of stout setae at sides; slightly curved in lateral view.

#### Food plants.

Saxifragaceae: *Itea parviflora* Hemsl.; Betulaceae: *Alnus formosana* (Burkill ex Forbes & Hemsl.) Makino; Symplocaceae: *Symplocos wikstroemiifolia* Hayata (Lee and Cheng 2010).

#### Distribution.

Endemic to Taiwan.

##### Key to Taiwanese species of the genus *Paleosepharia*

**Table:** 

1	Elytra black, with one pair of inwardly curved white stripes (Fig. 4D, F)	***P. nantouensis***
–	Elytra yellow, with black stripes along outer margins and transverse black bands	**2**
2	Only one transverse black band on elytra (Fig. 1A, 1C)	***P. amiana***
–	Two transverse black bands on elytra	**3**
3	Transverse black bands on elytra slender and weakly margined; tibiae reddish brown (Fig. 1D, F)	***P. excavata***
–	Transverse black bands on elytra broad and distinctly margined; tibiae black	**4**
4	Anterior transverse black bands reduced near suture, cluster of stout setae behind scutellum present in males (Fig. 7A); elytra straight, with posterior transverse black band continuously arcuate (Fig. 7A, C)	***P. yasumatsui***
–	Anterior transverse black bands connected and lacking cluster of stout setae behind scutellum in males (Fig. 4A); elytra rounded, with posterior transverse black bands separately arcuate by suture (Fig. 4A, C)	***P. formosana***

## Discussion

All species considered in this study definitely belong within *Paleosepharia*. This conclusion is based on the presence of subscutellar incised ridges on the elytra in males and the continuous elytral epipleurae and truncate apices. In males, the aedeagi of all species possess one paired cluster of short, basally hooked setae and one pair of elongate rows of slender and apically tapering lateral setae. Additionally, they possess two pairs of strongly sclerotized spiculae as Rizki et al. (2016) stated. An additional pair of elongate rows of large hooked spiculae from the apex to midpoint of the spiculae complex are found in males of *P. formosana*, *P. excavata*, and *P. yasumatsui*. An additional pair of extremely long, slender and apically curved speculae are found in males of *P. amiana* and *P. nantouensis*. However, the two moveable spines formed by the eighth tergites are not found in these Taiwanese species. Wagner and Bieneck (2012) speculated that the apically incised tectum is good character to separate *Paleosepharia* from other genera, but in Taiwanese species part of them (*P. excavata*, *P. formosana*, and *P. yasumatsui*) share this character. This character is not special for *Paleosepharia*. Additionally, the shapes of male aedeagi in *Paleosepharia* are characteristic: they recurve dorsally, not ventrally as in members of *Monolepta*. Female genitalic characters are similar and less diagnostic for species identification. However, the shape and number of bursal sclerites are unique and may prove to be synapomorphic for the genus.

## Supplementary Material

XML Treatment for Paleosepharia amiana

XML Treatment for Paleosepharia excavata

XML Treatment for Paleosepharia formosana

XML Treatment for Paleosepharia nantouensis

XML Treatment for Paleosepharia yasumatsui
